# Intravascular Large B-Cell Lymphoma: A Difficult Diagnostic Challenge

**DOI:** 10.1177/2324709614526702

**Published:** 2014-03-06

**Authors:** Maria S. Khan, Mark McCubbin, Sucha Nand

**Affiliations:** 1 The Ohio State University Medical Center, Columbus Ohio; 2 Loyola University Medical Center, Maywood, Illinois

**Keywords:** B cell, lymphoma

## Abstract

*Case Presentation*. A 69-year-old Hispanic male, with a past history of diabetes and coronary disease, was admitted for fever, diarrhea, and confusion of 4 weeks duration. Physical examination showed a disoriented patient with multiple ecchymoses, possible ascites, and bilateral scrotal swelling. Hemoglobin was 6.7, prothrombin time (PT) 21.4 seconds with international normalized ratio 2.1, partial thromboplastin time (PTT) 55.6 seconds, fibrin split 10 µg/L, and lactate dehydrogenase (LDH) 1231 IU/L. Except for a positive DNA test for Epstein–Barr virus (EBV) infection, extensive diagnostic workup for infections, malignancy, or a neurological cause was negative. Mixing studies revealed a nonspecific inhibitor of PT and PTT but Factor VIII levels were normal. The patient was empirically treated with antibiotics but developed hypotension and died on day 27 of admission. At autopsy, patient was found to have intravascular diffuse large B-cell lymphoma involving skin, testes, lung, and muscles. The malignant cells were positive for CD20, CD791, Mum-1, and Pax-5 and negative for CD3, CD5, CD10, CD30, and Bcl-6. The malignant cells were 100% positive for Ki-67. *Discussion*. Intravascular large cell B-cell lymphoma (IVLBCL) is rare form of diffuse large B-cell lymphoma and tends to proliferate within small blood vessels, particularly capillaries and postcapillary venules. The cause of its affinity for vascular bed remains unknown. In many reports, IVLBCL was associated with HIV, HHV8, and EBV infections. The fact that our case showed evidence of EBV infection lends support to the association of this diagnosis to viral illness. The available literature on this subject is scant, and in many cases, the diagnosis was made only at autopsy. The typical presentation of this disorder is with B symptoms, progressive neurologic deficits, and skin findings. Bone marrow, spleen, and liver are involved in a minority of patients. Nearly all patients have elevated LDH, and about 65% are anemic. About 20% have hepatic and renal dysfunction. The treatment consists of systemic chemotherapy with cyclophosphamide, doxorubicin, vincristine, prednisone plus rituximab (CHOP-R) and central nervous system prophylaxis. Retrospective data suggests that, with treatment, 51% to 82% of the patients achieve a complete remission and 27% to 56% are alive at 2-year follow-up. *Conclusion*. IVLBCL is a difficult diagnosis to make as the disease remains confined to the vascular lumen. It may be associated with certain viral illnesses, and this association needs to be explored further. It is important to consider this diagnosis in the appropriate settings because patients may achieve durable remissions with therapy.

## Background

Intravascular large cell B-cell lymphoma (IVLBCL) is a malignancy confined to the lumina of small blood vessels without any extravascular tumor mass or circulating lymphoma cells in the peripheral blood or bone marrow.^1^ IVLBCL is rare, and as of 2007, only about 300 cases have been reported, mostly involving B cells (IVLBCL). We report a case of IVLBCL in a patient presenting as fever of unknown origin.

## Case Presentation

A previously healthy 69-year-old Hispanic male presented with fever, diarrhea, anasarca, and confusion during the past 4 weeks. Physical examination showed a disoriented patient with multiple ecchymoses, ascites, scrotal swelling, and lower extremity edema. Hospital course was complicated by worsening renal failure, increasing oxygen requirements, spontaneous retroperitoneal bleed, and lactic acidosis. The patient was empirically treated with broad-spectrum antibiotics but developed altered mental status and circulatory collapse. He was transferred to the intensive care unit after developing respiratory failure and shock. He was intubated and placed on mechanical ventilation and started on vasopressor support. The patient subsequently expired on hospital day 27.

## Methods and Findings

**Figure 1.**
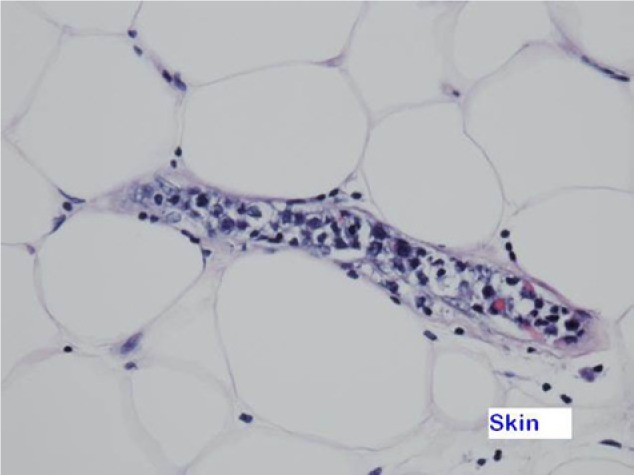
Skin biopsy.

Laboratory studies on admission showed white blood cell count 3800 µL with 62% segs and 24% lymphocytes, hemoglobin 6.7 g/dL, platelet count 117 × 10^6^/µL, blood urea nitrogen 44 mg/dL, creatinine 2.0 mg/dL, prothrombin time (PT) 21.4 seconds, international normalized ratio (INR) 2.1, partial thromboplastin time (PTT) 55.6 seconds, fibrinogen 592 mg/dL, fibrin split products 10 µg/L, and lactate dehydrogenase (LDH) 1231 IU/L. Mixing studies corrected the PT but not PTT. Factor VIII levels were normal. Total bilirubin was 1.0 mg/dL, direct bilirubin 0.4 mg/dL, haptoglobin level was normal at 73, aspartate aminotransferase 53 U/L (14-45 U/L), and alanine aminotransferase 17 U/L (15-40 U/L). Except for a positive DNA test for Epstein–Barr virus (EBV) infection, an extensive diagnostic workup for infections (HIV, arbo virus, viral hepatitis, quantiferon gold, histoplasma antigen, bacterial, fungal, AFB, pleural fluid analysis), malignancy (computed tomography scan of brain, chest, abdomen, pelvis, scrotal ultrasound, bone marrow biopsy), rheumatic diseases (ANA, ANCA, and C3/C4), and neurological causes (lumbar puncture) was negative. EBV was positive. Gallium scan showed uptake in the lung and groin but no focal lesions. At autopsy, he was found to have intravascular diffuse large B-cell lymphoma involving skin, testes, lung, and muscles (see Figure 1). The malignant cells were positive for CD20, CD791, Mum-1, and Pax-5 and negative for CD3, CD5, CD10, CD30, and Bcl-6. The malignant cells were 100% positive for Ki-67.

## Discussion

This patient died of a rare variant of diffuse large B-cell lymphoma, the commonest lymphoma seen in human beings. Despite a strong suspicion and extensive investigations, which included biopsies, a diagnosis could not be made in the ante-mortem period.

IVLBCL, also known as angiotropic large cell lymphoma,^1,2^ was first described in 1959 by Pfleger and Tappeiner.^3^ It was formerly known as “malignant angioendotheliomatosis.”^1^ It is a rare subtype of extranodal diffuse large B-cell lymphoma resulting from proliferation of neoplastic mononuclear cells within small blood vessels,^3^ particularly capillaries and postcapillary venules. The neoplastic cells are usually of B-cell origin but rarely can be T-cell or histiocytic derivation. The cause of its affinity for the vascular bed remains unknown. In many reports, IVLBCL was associated with HIV, HHV8, and EBV infections. It occurs primarily in adults (median age 67 years),^4^ with male-to-female ratio of 1:1.

The most commonly involved sites in this lymphoma are skin and brain, but it can involve any organ.^5^ It has 2 major subtypes: “Western” subtype with skin and neurologic involvement and “Asian” subtype that presents as a hemophagocytic syndrome with multiorgan involvement. A “cutaneous” variant has also been described wherein the disease remained confined to the skin and has a favorable prognosis.^6^

The typical presentation of this disorder is with B symptoms (fever, night sweats, and weight loss); progressive neurologic deficits,^7,8^ such as dementia, headaches, seizures, and focal deficits; and skin lesions, which have been described as red, tender plaques or ulcerations mostly on lower extremities with edema.^9^ The skin lesions can be misinterpreted as thrombophelebitis, erythema nodosum, or erysipelas.^9,10^

Diagnosis is made when large lymphoma cells are seen within small to medium-sized blood vessels. This often requires obtaining multiple skin^11^ or other biopsies. In general, the IVLBCL does not involve bone marrow, lymph nodes, peripheral blood, and cerebrospinal fluid. The role of imaging is limited, as the tumor does not present as a mass lesion. Similarly, positron emission tomography scan, normally a useful tool in diagnosis and monitoring of lymphomas, has a limited role in IVLBCL unless the patient has central nervous system (CNS) involvement.^4,12^

Treatment consists of systemic chemotherapy with cyclophosphamide, doxorubicin, vincristine, prednisone plus rituximab (CHOP-R) and CNS prophylaxis. Retrospective data suggests that, with treatment, 51% to 82% of the patients achieve a complete remission and 27% to 56% are alive at 2-year follow-up.^13^

Untreated IVLBCL has an extremely aggressive course and poor prognosis, with a median survival of 5 months.^14-17^

## Conclusion

Look for uncommon variants and locations of the suspected disorder when the data suggest a particular diagnosis. In this patient, a high LDH level suggested a hematologic malignancy as other possibilities had been ruled out. Even though all his biopsies were negative for lymphoma even on retrospective review, a more extensive examination may have yielded the diagnosis of a potentially curable disease.

## References

[1] Shimada K Kinoshita T Naoe T Nakamura S Presentation and management of intravascular large B-cell lymphoma. Lancet Oncol. 2009;10:895-902. 19717091 10.1016/S1470-2045(09)70140-8

[2] Bhawan J Wolff SM Ucci AA Bhan AK Malignant lymphoma and malignant angioendotheliomatosis: one disease. Cancer. 1985;55:570-576. 3880661 10.1002/1097-0142(19850201)55:3<570::aid-cncr2820550316>3.0.co;2-0

[3] Pfleger L Tappeiner J On the recognition of systematized endotheliomatosis of the cutaneous blood vessels (reticuloendotheliosis). Hautarzt. 1959;10:359-363. 14432547

[4] Kiyohara T Kumakiri M Kobayashi H Shimizu T Ohkawara A Ohnuki M A case of intravascular large B-cell lymphoma mimicking erythema nodosum: the importance of multiple skin biopsies. J Cutan Pathol. 2000;27:413-418. 10955689 10.1034/j.1600-0560.2000.027008413.x

[5] Murase T Yamaguchi M Suzuki R Intravascular large B-cell lymphoma (IVLBCL): a clinicopathologic study of 96 cases with special reference to the immunophenotypic heterogeneity of CD5. Blood. 2007;109:478-485. 16985183 10.1182/blood-2006-01-021253

[6] Bogomolski-Yahalom V Lossos IS Okun E Sherman Y Lossos A Polliack A Intravascular lymphomatosis—an indolent or aggressive entity? Leuk Lymphoma. 1998;29:585-593. 9643572 10.3109/10428199809050918

[7] Swerdlow SH Campo E Harris NL WHO Classification of Tumours of Haematopoietic and Lymphoid Tissues. 4th ed. Lyon, France: IARC Press; 2008.

[8] Chapin JE Davis LE Kornfeld M Mandler RN Neurologic manifestations of intravascular lymphomatosis. Acta Neurol Scand. 1995;91:494-499. 7572046 10.1111/j.1600-0404.1995.tb00452.x

[9] Glass J Hochberg FH Miller DC Intravascular lymphomatosis. A systemic disease with neurologic manifestations. Cancer. 1993;71:3156-3164. 8490846 10.1002/1097-0142(19930515)71:10<3156::aid-cncr2820711043>3.0.co;2-o

[10] Perniciaro C Unusual cutaneous lymphomas. Dermatol Surg. 1996;22:288-292. 8599741 10.1111/j.1524-4725.1996.tb00320.x

[11] Roglin J Boer A Skin manifestations of intravascular lymphoma mimic inflammatory diseases of the skin. Br J Dermatol. 2007;157:16-25. 17506787 10.1111/j.1365-2133.2007.07954.x

[12] Shimada K Murase T Matsue K Central nervous system involvement in intravascular large B cell lymphoma: a retrospective analysis of 109 patients. Cancer Sci. 2010;101:1480-1485. 20412122 10.1111/j.1349-7006.2010.01555.x PMC11158344

[13] Yamada S Nishii R Oka S FDG-PET a pivotal imaging modality for diagnosis of stroke-onset intravascular lymphoma. Arch Neurol. 2010;67:366-371. 20212240 10.1001/archneurol.2010.6

[14] Shimada K Matsue K Yamamoto K Retrospective analysis of intravascular large B-cell lymphoma treated with rituximab-containing chemotherapy as reported by the IVL study group in Japan. J Clin Oncol. 2008;26:3189-3195. 18506023 10.1200/JCO.2007.15.4278

[15] Ponzoni M Ferreri AJ Campo E Definition, diagnosis, and management of intravascular large B-cell lymphoma: proposals and perspectives from an international consensus meeting. J Clin Oncol. 2007;25:3168-3173. 17577023 10.1200/JCO.2006.08.2313

[16] Domizio P Hall PA Cotter F Angiotropic large cell lymphoma (ALCL): morphological, immunohistochemical and genotypic studies with analysis of previous reports. Hematol Oncol. 1989;7:195-206. 2651272 10.1002/hon.2900070303

[17] Dufau JP Le Tourneau A Molina T Intravascular large B-cell lymphoma with bone marrow involvement at presentation and haemophagocytic syndrome: two Western cases in favour of a specific variant. Histopathology. 2000;37:509-512. 11122432 10.1046/j.1365-2559.2000.00980.x

